# The prognostic value of tumor-stromal ratio combined with TNM staging system in esophagus squamous cell carcinoma

**DOI:** 10.7150/jca.50439

**Published:** 2021-01-01

**Authors:** Ruyuan He, Donghang Li, Bohao Liu, Jie Rao, Heng Meng, Weichen Lin, Tao Fan, Bo Hao, Lin Zhang, Zilong Lu, Haojie Feng, Ziyao Zhang, Jingping Yuan, Qing Geng

**Affiliations:** 1 Department of Thoracic Surgery, Renmin Hospital of Wuhan University, Wuhan, China; 2 Department of Pathology, Renmin Hospital of Wuhan University, Wuhan, China

**Keywords:** ESCC, tumor-stromal ratio, TNM staging system, prognosis, pathology

## Abstract

**Background:** Tumor stroma is a crucial component of the tumor environment that interacted with tumor cells and modulated tumor cell proliferation, immune evasion, and metastasis. Tumor-stromal ratio (TSR) has been confirmed as an influential independent prognostic factor for diverse types of cancer, but it was seldom discussed in esophagus squamous cell carcinoma (ESCC).

**Methods:** In present study, pathological sections from the most invasive part of the ESCC of 270 patients were analyzed for their TSR by visual inspection and software. The TSR was combined with the TNM staging system to further explain its predictive value of prognosis. The 57 cases ESCC from TCGA database also were included as an independently validated cohort.

**Results:** Our results indicated that TSR was a robust prognostic factor for ESCC patients. TSR by visual inspection was dependable to reflect the stroma percent of the tumor compared to software calculation. Compared with stroma-low groups, the risk of death increased by 153.1% for patients in the stroma-high group [HR=2.531 (95%CI 1.657-3.867), *P*<0.001]. The results of ROC analysis in two cohorts indicated that TSNM staging system had better resolving ability with the largest area under the curve [0.698 95%CI (0.635-0.760), 0.691 95%CI (0.555-0.807)], compare to TNM. The novel TSNM staging system revealed strong predictive performance (*P*<0.001).

**Conclusion:** TSR was a reliable dependent indicator for ESCC prognosis. The TSNM staging system has a better discriminative ability than the conventional TNM staging system, especially for III stage patients.

## Introduction

Esophageal cancer is the sixth most common cause of cancer death globally^1^. Esophageal squamous cell carcinoma (ESCC) is the most common histological subtype of esophageal cancer, particularly in areas of eastern Asia, eastern and southern Africa. Approximately 90% esophageal cancer cases are ESCC in China where the disease is a major public health problem^1^-^3^.

Nowadays, there is a comprehensive understanding of tumor indicating that attentions are required not only to tumor cells but also to the tumor microenvironment (TME), which contains diverse cell populations, signaling factors, and structural molecules^4^. As the main elements of TME, tumor stroma had a dynamic interaction with tumor cells that can influence tumor growth, metastasis and chemoresistance^5^,^6^. Many studies showed that some essential components of TME such as cancer-associated fibroblasts (CAFs) and immune cells, including tumor-associated macrophages (TAMs) regulatory T cells (Tregs) myeloid-derived suppressor cells (MDSCs, play a significant role in the initiation, progression, immune evasion and survival of tumor^7^-^10^. Although tumor stroma is a crucial factor that interacted with tumor cells and support all stages of tumorigenesis, it has not been integrated in routine clinical decision making yet. A parameter that represent the amount of tumor-associated stroma is the tumor-stroma ratio (TSR), which has been described as a potential indicator of prognosis for various solid cancer types. TSR was initially found to be significantly associated with clinical prognosis in colon cancer^11^. Nowadays, the predictive value of TSR has been extensively estimated in various cancers, including colon cancer^12^-^16^, breast cancer^17^-^21^, gastric cancer^22^-^24^, and esophageal adenocarcinoma^25^,^26^. What's more, some oncologist applied TSR to the prediction of adjuvant chemotherapy effect in colon cancer^27^,^28^. TSR was also combined with TNM staging of gastric cancer to be creatively proposed as the TSNM staging system and performed good prediction^24^. In summary, TSR is a strong independent prognostic tool for diverse kinds of cancers, and might be integrated with the TNM staging system in the future.

The prognosis of ESCC is strongly related to TNM staging. Currently, there were not enough evidence to evaluate the predicted value of TSR within detailed TNM staging of ESCC. How to combine TSR and traditional TNM staging to better predict patient prognosis also requires further exploration. At present study, we adopt innovative methods to verify the reliability of the visual assessment for TSR, which was ignored in almost all studies of TSR generally. We proved that TSR can robustly predict the prognosis of patients with different staging ESCC, especially for those of stage II and III. The TSNM staging, integrating TSR with TNM staging, had a better discriminative ability than the single TNM staging in ESCC.

## Materials and methods

### Study population and characteristic

All records of the patients were retrieved, who underwent esophagectomy at the Department of Thoracic Surgery of Renmin Hospital of Wuhan University from January 2010 to August 2014. The information of the participants involved major clinical and demographic characteristics, pathological characteristics including histologic grade, depth of invasion, number of lymph nodes and number of lymph nodes with metastases. The inclusions: (1) Patients with esophageal cancer who underwent radical esophagectomy. (2) Pathological diagnosis is ESCC. The exclusion: (1) Receive neoadjuvant chemotherapy (2) Distant metastasis (3) The medical records and survival data were incomplete (4) The pathological sections were unavailable. (5) Complicating other cancer. Eventually we included 270 patients, with their clinical and pathological information shown in table 1. The study was approved by the Institute Research Medical Ethics Committee of Renmin Hospital of Wuhan University. The 7th edition UICC/AJCC TNM system was used to determine the TNM stages. The primary endpoint was death, and patients who were alive at the last follow-up or lose of follow up were recorded as censored events. Overall survival (OS) was used to evaluate the prognosis, and was defined as the duration from esophagectomy to death or the last follow-up. In addition, we also acquired the data of 95 cases ESCC patients from TCGA databases. 38 cases were excluded because usable pathological images are not available. The remaining 57 cases were used to validated our conclusion as an independent cohort.

### Histopathological evaluation

**Figure 1:**
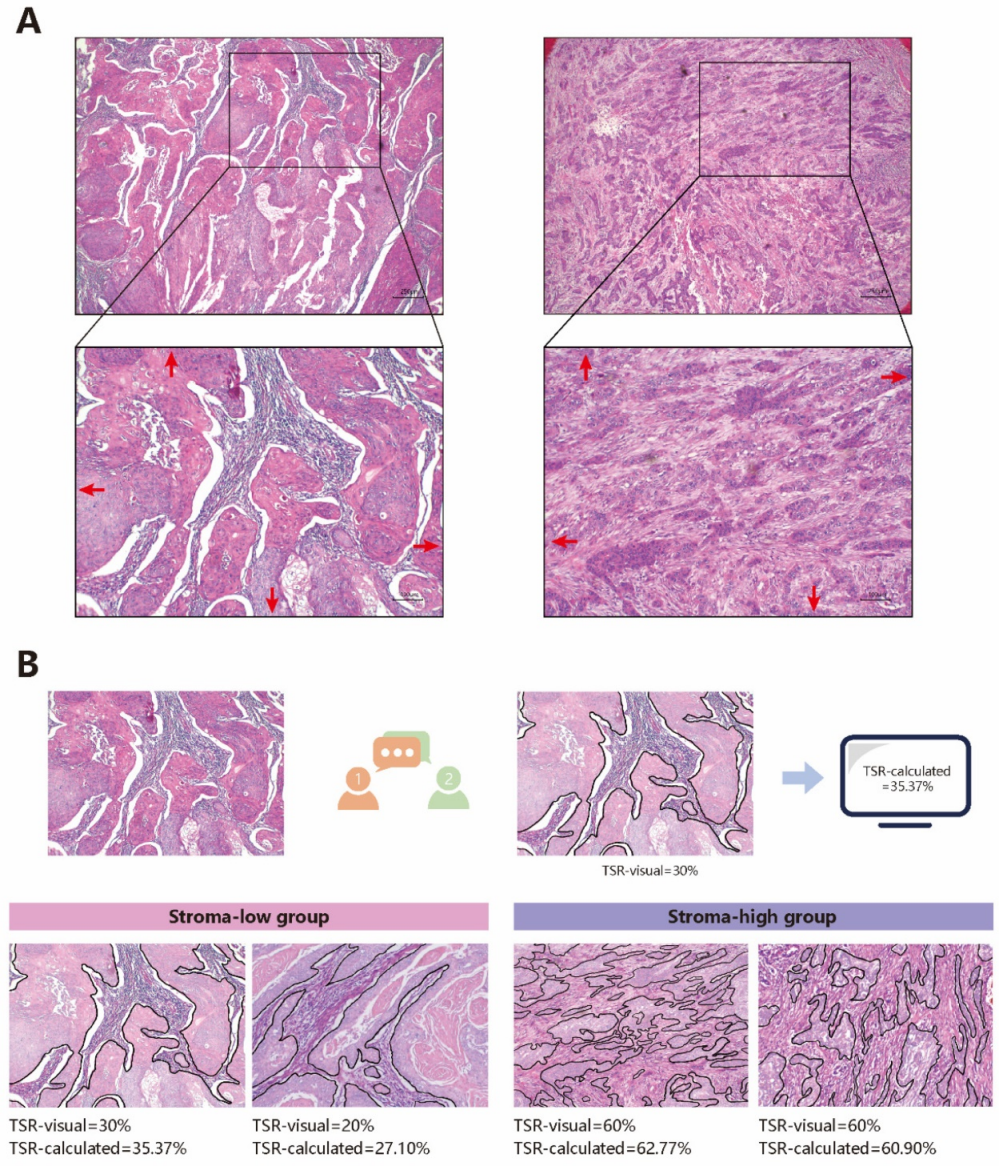
**The evaluation of TSR**. A Choose appropriate field to estimate TSR. B The Process of evaluation of TSR and typical stroma

The evaluations were performed as published studies^24^,^29^. All tissue samples were retrieved from the pathology archives, and 5 μm Haematoxylin and eosin (H&E) stained histologic sections were microscopically analyzed. The most invasive section, which was used to confirm T staging, was selected under a 4× objective. Subsequently, a 10× objective was used to select a field where both stroma and tumor were present and tumor cells were visualized at all borders of the image field from the most invasive sections (Fig 1A). Although the visual evaluation of TSR was convenient, it had the disadvantages of instability and poor repeatability. A previous study showed that visually assessed TSR may be not serve as an independent prognostic factor in multivariate analysis^30^. Thus, we took an additional method to test the accuracy of visual assessment. The method consisted of two steps:(1) Two investigators outlined the tumor area on selected image field, and highlight this area. The two investigators visually assessed the proportion of the stroma area, using 10% increments. Eventually, the stroma area considered most suitable for TSR assessment was identified during a consensus meeting between the two investigators in which consensus stroma area and score were determined, and the score was recorded as TSR-visual. (2) ImageJ (version 1.51), an open source image processing program developed at the National Institutes of Health and the Laboratory for Optical and Computational Instrumentation^31^,^32^, was used to calculate the percentage of stroma area. The calculation result was recorded as TSR-calculated (Fig 1B).

### Statistical analysis

In this study, SPSS software (version 21.0) and MedCalc software (version 19.5) were used to perform statistical analysis. ImageScope (version 12.4) was used to analyze the digital pathological slide. Cohen's Kappa (κ) was used to calculate inter-observer agreements. Mann-Whitney U test and Pearson χ^2^ test was performed to compare qualitative variables. Life-table was used for survival analysis and the log rank test was used to calculate the significance among patients' subgroups. The Cox regression model was used for multivariate analysis. The predictive value of the TSR was performed by receiver operating characteristic curve (ROC) analysis. The results were considered statistically significant at *P*<0.05.

## Results

### Patients and clinicopathological features

In this study, a total of 270 patients were enrolled, including 222 (82.2%) males and 48 (18.8%) females. There were 31 (11.5%), 134 (49.6%) and 105 (38.9%) patients with tumor in the upper, middle and lower part of esophagus, respectively. No patients had distant metastases, there were 62 (23.0%), 140 (51.9%) and 68 (25.2%) patients with stage I, stage II and stage III cancer, respectively. Major clinicopathological features were shown in Table 1.

### The evaluation of TSR and its relationship with patients' clinicopathological features

**Figure 2:**
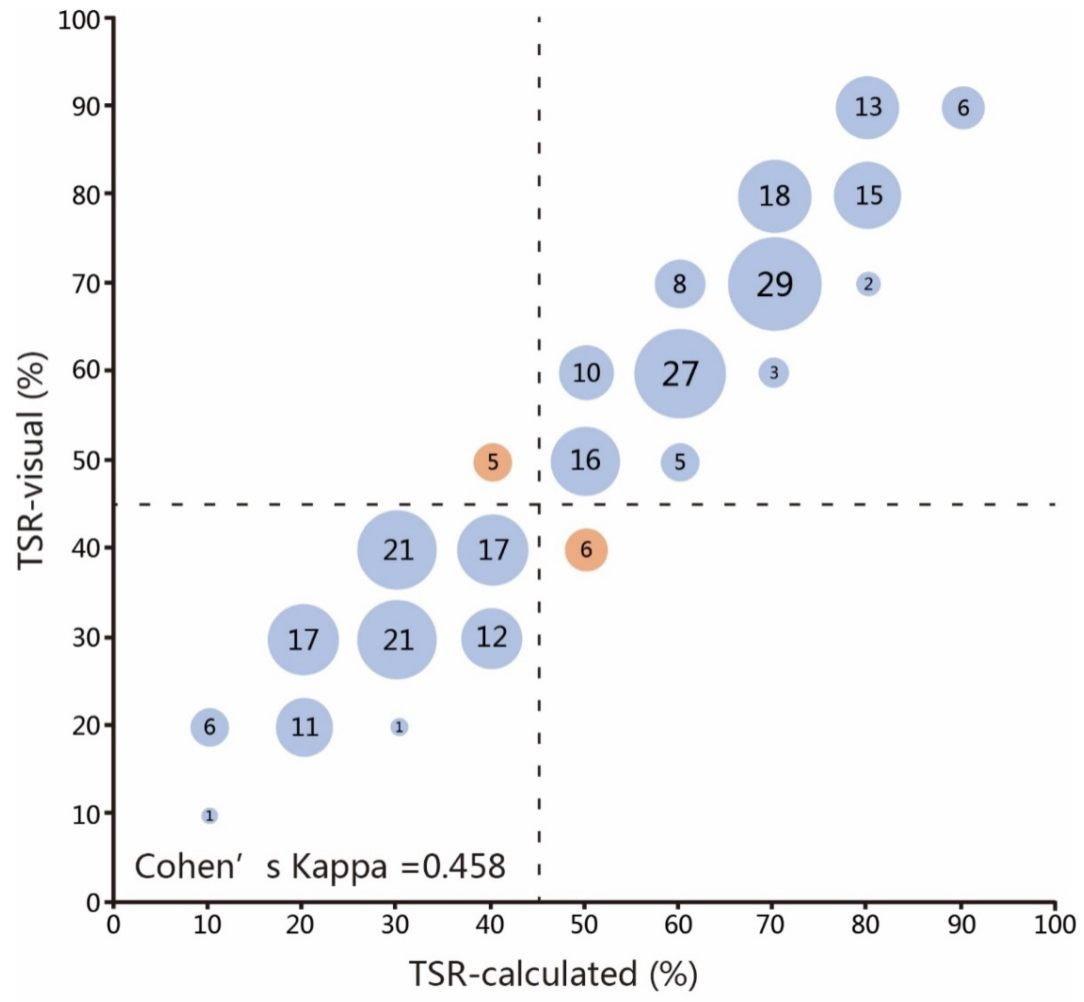
**Scatter plot of assessed stroma percentages in 270 patients for pathologists and software.** The co-occurrence of assessed percentages was indicated by circles with areas proportional to the amounts of patients scored with the corresponding TSR value. The dashed lines represent the boundary between stroma-low and stroma-high group according to the 50% cut-off value. Red circles indicate cases where pathologists and software disagreed (11 in total). Evaluation of 127 (47.03%) cases were not coincide exactly.

We performed the evaluation of the TSR successfully in all 270 patients. We innovatively used more accurate methods to evaluate the TSR. The median TSR-visual was 0.5 and the mean of TSR-visual were 0.53. The median TSR-calculated was 0.55 and the mean of TSR-calculated was 0.57. The cut-off point was set at 0.50, which was in an almost perfect agreement with the cut-off points of other cancers^33^. TSR-visual and TSR-calculated were divided into two subgroups: a 'stroma-low' subgroup and a 'stroma-high' subgroup, based on a cut-off value. A moderate agreement between the TSR-visual and TSR-calculated (κ =0.458) was reached on the basis of the 0.5 cut-off point (Fig. 2). While the evaluation of 127 (47.03%) cases were not coincident exactly, only in 11 (4.07%) cases did the disagreement exist in the dividing of patients into stroma-low or stroma-high groups. The visual evaluation performed great accuracy. The TSR-visual were dependable to reflect the stroma percent of tumor, and was used to perform subsequent analysis. Out of the 270 analyzed samples, 113 (41.86%) patients were classified as stroma-low and 157 (58.15%) patients were classified as stroma-high. The clinicopathological features associated with TSR were shown in Table 1. There was a correlation between TSR and histological grade (*P*=0.046), and no significant differences regarding age, gender, tumor location and TNM stage were observed.

### Survival analysis and multivariate analysis

In the present study, the median OS was 43 (range 1-113) months. Many clinicopathological factors, including age, histological classification, lymph node (pN) status, serosa invasion (pT) status, TNM stage, were associated with OS (*P*<0.05 for all). The estimated 3-year and 5-year survival rates (%) in stoma-low group were 80.46% and 61.33%. The estimated 3-year and 5-year survival rates (%) in stoma-high group were 61.43% and 40.61%. There was a significant difference between stroma-low and stroma-high groups in OS (Table 2).

The results of univariate and multivariate analyses demonstrated that age, histological grade, TNM stage, and TSR were independent prognostic factors for OS (*P*<0.05 for all). Compared to stroma-low groups, the risk of death increased by 153.1% for patients in the stroma-high group [HR=2.531 (95%CI 1.657-3.867), *P*<0.001] (Table 3).

### The association of TSR with TNM stage

**Figure 3:**
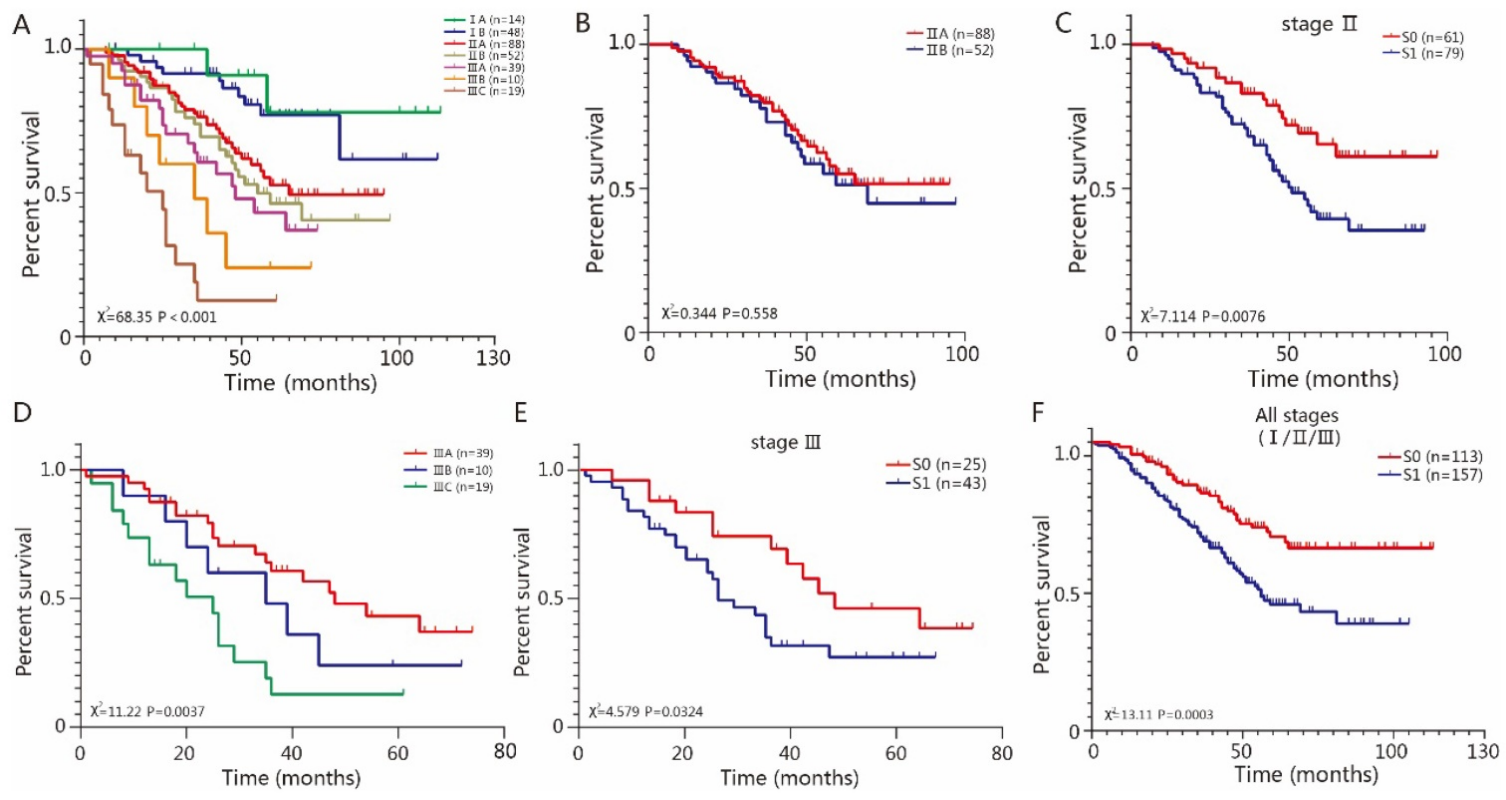
**The association of TSR with TNM staging system.** A TNM staging system were heterogeneous. B Among the stage II, there was no significant difference between IIA and IIB (*P*=0.558). C There was significant difference between group S0 and S1 (*P*=0.0076). D Among the stage III, there was significant difference between IIIA, IIIB and IIIC (*P*=0.0037). E There was significant difference between group S0 and S1 (*P*=0.0324). F There were significant difference between S0 and S1 regarding all stage (*P*=0.0003).

Our results have shown that TSR is a strong dependent parameter for ESCC prognosis. We regarded the TSR as an indicator for TME and focused on the discriminative ability of TSR. We termed stroma-low as S0 and stroma-high as S1 and performed group analysis on the basis of the TNM stage and TSR. TNM staging systems showed good discriminative ability in patients from stage I to stage III. (*P*<0.001) (Fig 3A). Among the stage I patients, there was no significant difference between stage IA and IB (*P*=0.7063), and there was no significant difference between group S0 and S1 (*P*=0.3299). Among the stage II patients, there was no significant difference between II A and IIB (*P*=0.558) (Fig 3B), while there was a significant difference between group S0 and S1 (*P*=0.0076) (Fig 3C). Among the stage III patients, there were significant differences between IIIA, IIIB and IIIC (*P*=0.0037) (Fig 3D), there was also a significant difference between group S0 and S1 (*P*=0.0324) (Fig 3E). The TSR showed dependable discriminative ability among stage II and III patients. As expected, there were significant differences between S0 and S1 in all stages (I/II/III) (Fig 3F), due to the fact that stage II and III patients were dominated in our cohorts. In addition, we also validated the predictive value of TSR in another cohort containing 57 cases from TCGA. For patients of all stages, there was a significant difference between group S0 and S1 (*P*=0.0023) (Fig S1A). We also analyzed the discriminative ability of TSR in II stage, which is the dominated stage in this cohort. Our results indicated that there was no significant difference between II A and IIB (*P*=0.478) (Fig S1B), while there was a significant difference between group S0 and S1 (*P*=0.0316) (Fig S1C).

### The combination of TSR and TNM staging system

**Figure 4:**
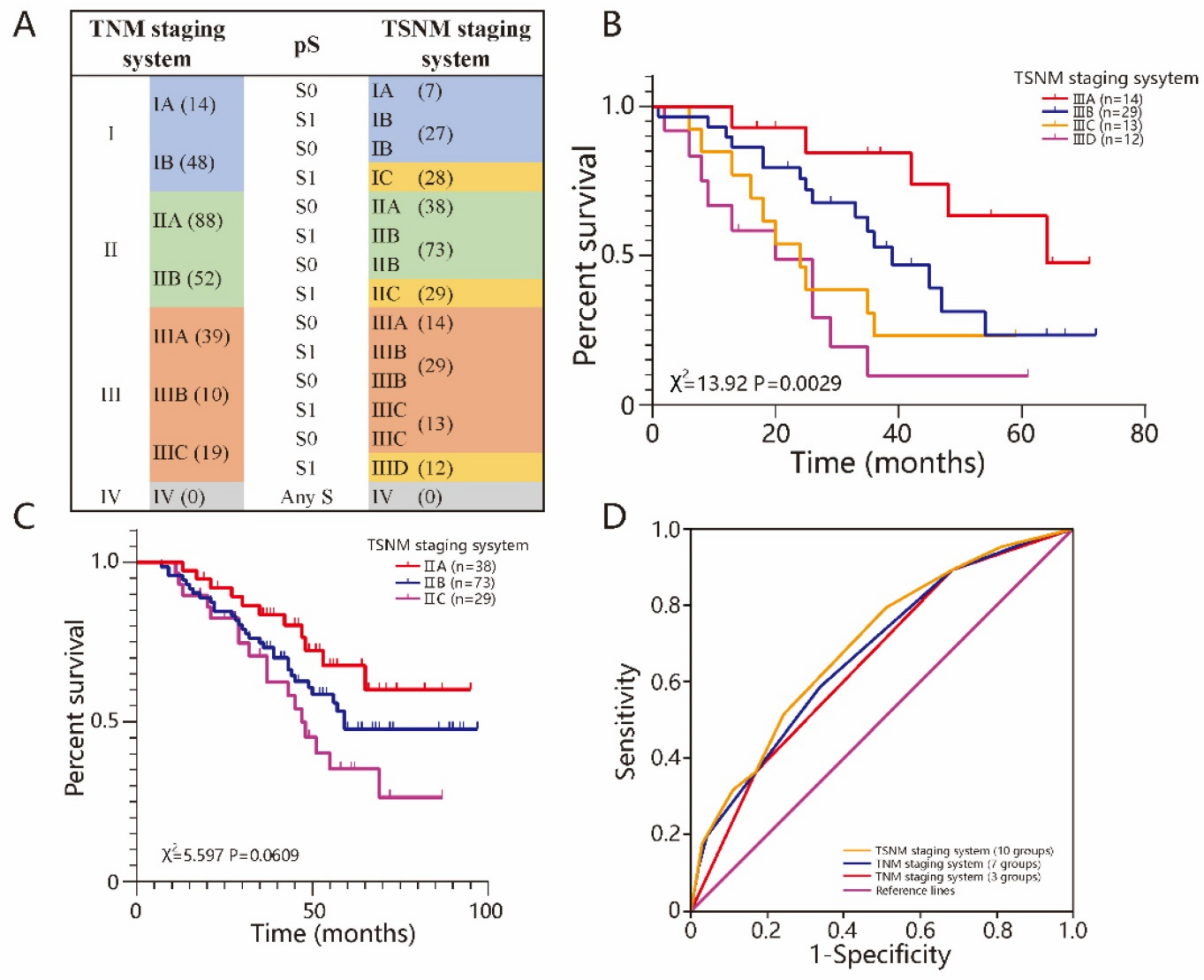
**The combination of TSR and TNM staging system.** A The TSNM staging system, based on 7th TNM staging system and pS status. The number represents the number of people who belong to the staging. B Among the stage III, there was significant difference between IIIA, IIIB, IIIC, IIID (*P*=0.0029). C Among the stage II, there was no significant difference between IIA, IIB and IIC (*P*=0.0609). D TSNM performed well in predicting the clinical outcomes of ESCC patients compared to other factors

Previous studies termed TSR as pS (pathological stromal status), which was similar to pT and pN, and integrated it into the TNM staging system. The new staging system was called the TSNM (tumor-stroma node metastasis) staging system^24^. The novel TSNM staging system had 3 new subgroups named IC, IIC and IIID compared to the TNM stage system. The patients in the S0 group stayed in the same stage in the novel system as they did in the TNM system, but those in the S1 group were in a higher stage (Fig 4A). Our results showed that the TSNM staging system only had significant differences among stage III group patients (*P*=0.0029) (Fig 4B), and had no significant difference among stage I and II patients (Fig 4C) (*P*>0.05 for all). ROC analysis was used to further test the predictive value of the TSNM staging system (Fig 4D). The TSNM staging system had a robust predictive value with a large area under the curve [0.698 95%CI (0.635-0.760)]. The areas under the curve were 0.676 [95%CI (0.612-0.740)] for 4 groups and 0.653 [95%CI (0.593-0.710)] for another 7 in the TNM staging system, respectively. There were significant differences between the TSNM staging system and the TNM staging system (*P*=0.006 for 4 groups and *P*=0.0254 for another 7). The patients from cohort of TCGA also was reclassified according to TSNM stage (Fig S1D). The results also suggested that TSNM was a robust predictive staging system with a largest area under the curve [0.691 95%CI (0.555-0.807)] (Fig S1E). In conclusion, the TSNM has a better discriminative and predictive ability.

## Discussion

As an important part of the tumor microenvironment, the stroma plays an inseparable role in modulating immune function, promoting angiogenesis, and inducing metastasis. More and more studies have focused on tumor stroma besides tumor cells. However, there were few TME parameters applied to tumor diagnosis and therapy. Currently, many previous studies have demonstrated that TSR was a strong independent prognostic factor for several cancers. Although TSR only roughly reflected the stroma percent of TME, the evaluation of TSR was a quick and convenient process that can be performed during pathological examination without additional costs above standard diagnostics. It is a simple, relatively inexpensive morphometric measurement^24^.Therefore, it was reported that the TNM Evaluation Committee (UICC) and the College of American Pathologists (CAP) have discussed about TSR and acknowledged its potential for integration with the TNM staging system recently. To achieve this in colon cancers, some scientists are currently investigating the reproducibility of (visual) TSR assessment in a large European multi-center study^30^,^33^.

Our study applied the software to evaluate TSR innovatively and attempting to combine TSR with the TNM staging system. We paid attention to verify the reliability of the visual assessment for TSR, which was ignored in almost studies of TSR. The sample size of our study also was the biggest among the studies about TSR within esophageal cancer. Previous studies mainly pay attention to the TSR in esophageal adenocarcinoma (EAC)^34^,^35^. As we all know, esophageal adenocarcinoma and esophageal squamous carcinoma (ESCC) differ widely in prevalence, pathogenesis and prognosis. Our study mainly focuses on the predictive value of TSR in patients with different stages ESCC. TSR was universally assessed by pathologists visually on the percent of the stromal area and divided into two groups according to the cut-off value. Visual assessment is convenient but very subjective. Previous a study indicated that visually assessed TSR did not serve as an independent prognostic factor^30^, which may be associated with the assessment bias from pathologists. We innovatively use an efficient and accurate method which combined the visual assessment with software to calculate in order to verify reliability of the visual assessment method. As expected, our results indicated the visual evaluation and the computer-calculated area percentage have a margin of error of more than 10% in 127 (47.03%) cases. Although this result only had a minimal impact on the distinction between TSR high and low groups in our study, it proved that assessment bias from pathologists was indeed existing and the reliability of the visual assessment need to be tested in the future research on TSR. Our results proved that the methods of software calculation and visual assessment had an outstanding consistency, when based on a threshold of 50%. But there were still some deviations in evaluating the interval of TSR value accurately. Recently, researchers also have developed computer-aided quantification techniques for the evaluation of TSR in colon cancer, and the results showed that automated TSR-high group was found to be predictive of both disease-specific survival [hazard ratio=2.48 (95% confidence interval 1.29-4.78)] and disease-free survival hazard ratio=2.05 (95% confidence interval 1.11-3.78). With the development of artificial intelligence technology, in addition to simply evaluating the percent of stromal area, we can implement a discrimination of tumor stromal components and distinguish different histological types by training the deep learning algorithm. Graphical approaches are currently being used to evaluate the spatial arrangement and architecture of different types of tissue elements in order to predict clinical outcomes of the patients. Saltz et al. described the use of a convolutional neural network (CNN) in conjunction with the feedback from pathologists to automatically detect the spatial organization of tumor-infiltrating lymphocytes (TILs) in images of tissue slides from The Cancer Genome Atlas, and found that this feature was predictive of the outcomes for 13 different cancer subtypes^33^.

The patients included in our study were in stage II predominantly, and therefore had a higher three-year and five-year survival rate overall. Our results suggested that TSR had a robust predictive value for the prognosis of patients with stage II and III ESCC, which was consistent with the results of previous studies^24^^.^ However, some studies showed that while TSR has a predictive value in the prognosis of patients with stage I and II in esophageal adenocarcinoma, it has no discriminative ability between patients of stage III and IV^26^. In the present study, the OS of stage I patients in the stroma-high and the stroma-low group showed no significant differences. We speculated that the stroma may play a stronger influence for stage II, III patients by modulating tumor cell proliferation, immune evasion, and metastasis.

Currently, more and more studies have recognized the importance of tumor stroma for tumor development and prognosis. However, there was no parameter to evaluate the status of tumor stroma in the current TNM staging system yet. A parameter aimed to assess the tumor microenvironment was necessary in clinical work. Therefore, increasing studies had recognized the prognostic value of TSR. Previous studies have attempted to integrate the TSR into the TNM staging system, forming a new TSNM staging system. We verified the effect of this innovative staging system in our study. Although our results from two cohorts indicated that TSNM staging was indeed better than the traditional TNM staging in terms of sensitivity and specificity associated with prognosis, and the results were statistically significant, our results also indicated that the method of integrating pS into the TNM staging system directly, which was similar to pN or pT, may not greatly satisfied. The new TSNM staging had a satisfying discriminating ability for patients in stage III, but showed no statistical difference for patients in stage I and II. In addition, the new TSNM staging system was more complicated, and the number of subgroups increased. Therefore, we believed that this combining method was not perfect, some more innovative ways which can integrate the TSR into TNM staging system are necessary, such as the combined way between histological grade and the TNM staging system.

The reasons for the associations of high stroma with bad prognosis were discussed in many studies. A high stromal content was a reflection of the highly activated interaction between tumor and stromal cells. Maybe the tumor with more stroma were able to produce more stroma-derived growth factors thus increasing the overall tumor burden^36^. High proportion of stromal means that more stroma is activated by fewer cancer cells, indicating that the coevolution between cancer cells and tumor stroma is more effective, and consequently, results in poorer overall survival^24^. There were various mechanisms proposed to explain how the tumor stromal components contributed to tumor progression, invasion and metastasis. These contents were not discussed in this study detailedly.

There were also some limitations in this study. The pathological stages of the cohorts were mainly stage II, while lacking in stage IV, leading to the fact that the predictive ability of TSR for the prognosis of advanced ESCC patients was unknown. In addition, some follow-up was interrupted for various reasons, thus the 3-year and 5-year survival rates, calculated by life-table method, may be higher than the actual survival rate. Moreover, the sample size of the validated cohort from TCGA was not large enough and the results of it required a larger sample size multi-center study for further validation.

In conclusion, we applied the software to assist in the assessment of TSR and verified the reliability of the visual assessment in ESCC. Survival analysis and risk factor analysis showed that patients with low proportion of stroma had longer OS than those with high proportion of stroma, especially for those of stage II and III. We further integrated TSR into the TNM staging system and found that the new TSNM staging had a better discriminative ability than the traditional TNM staging system among the stage III group patients.

## Supplementary Material

Supplementary figure 1.

## Figures and Tables

**Table 1:** All patients clinicopathological features.

Items	Total *N* (%)	TSR<0.5 *N* (%)	TSR>0.5 *N* (%)	*P* value
Age (M±SD)	59.6 ± 8.40	59.1 ± 8.30	59.9 ± 8.45	0.541
Gender				
Male	222 (82.2)	94 (83.2)	128 (81.5)	0.725
Female	48 (18.8)	19 (16.8)	29 (18.5)	
Tumor location				
Upper	31 (11.5)	16 (14.2)	15 (9.6)	0.471
Middle	134 (49.6)	53 (46.9)	81 (51.6)	
Low	105 (38.9)	44 (38.9)	61 (38.9)	
pT status				
T1	26 (9.6)	14 (12.4)	12 (7.6)	0.507
T2	108 (40.0)	46 (40.7)	62 (39.5)	
T3	123 (45.6)	47 (41.6)	76 (48.4)	
T4	13 (4.8)	6 (5.3)	7 (4.5)	
T1/T2	134 (49.6)	60 (53.1)	74 (47.1)	0.334
T3/T4	136 (50.4)	53 (46.9)	83 (52.8)	
pN status				
N0	193 (71.5)	84 (74.3)	109 (69.4)	0.815
N1	38 (14.1)	15 (13.3)	23 (16.4)	
N2	24 (8.9)	9 (8.0)	15 (5.6)	
N3	15 (5.6)	5 (4.4)	10 (3.7)	
Histological grade				
high	145 (53.7)	52 (46.0)	93 (59.2)	**0.046**
middle	90 (33.3)	47 (41.6)	43 (27.4)	
low	35 (13.0)	14 (12.4)	21 (13.4)	
TNM stage				
Ⅰ	62 (23.0)	27 (23.9)	35 (22.3)	0.617
Ⅱ	140 (51.9)	61 (54.0)	79 (50.3)	
Ⅲ	68 (25.2)	25 (22.1)	43 (27.4)	
Ⅰ/Ⅱ	202 (74.9)	88 (77.9)	114 (72.6)	0.326
Ⅲ	68 (25.2)	25 (22.1)	43 (27.4)	

Bold indicates values with a significant difference *P*<0.05

**Table 2:** Analyses of factors regarding overall survival (OS)

Variables	*N*	3-year survival rate (%)	5-year survival rate (%)	Log-rank test χ value	*P* value
Gender					
Male	222	67.50	49.13	0.6756	0.411
Female	48	78.51	52.45		
Age (years)					
<60	137	79.08	57.94	10.79	0.001
≥60	133	59.66	41.14		
Location					
Upper	31	82.09	39.91	1.173	0.556
Middle	134	69.47	54.02		
Lower	105	66.11	47.08		
Histological grade					
1	145	79.00	57.86	14.13	0.001
2	90	57.32	41.33		
3	35	68.17	39.22		
pT status					
T1/T2	134	81.15	61.43	17.75	<0.001
T3/T4	136	57.60	36.52		
pN status					
N0	193	77.54	58.10	44.50	<0.001
N1	38	70.59	32.66		
N2	24	32.29	21.53		
N3	15	21.20	21.20		
TNM stage					
Ⅰ	62	91.40	76.83	35.24	<0.001
Ⅱ	140	72.15	48.36		
Ⅲ	68	42.47	24.75		
TSR					
stromal-low	113	80.46	61.33	13.11	<0.001
stromal-high	157	61.43	40.61		

Bold indicates values with a significant difference *P*<0.05

**Table 3:** Univariate and multivariate analyses of factors associated with overall survival (OS)

Factors	Univariate analysis			Multivariate analysis		
	HR	95%CI	*P* value	HR	95%CI	*P* value
Gender						
Female	1.000			1.000		
Male	1.234	0.7701-1.976	0.411	1.042	0.611-1.777	0.880
Age (years)						
<60	1.000			1.000		
≥60	1.847	1.270-2.685	0.001	2.153	1.452-3.192	**<0.001**
Location						
Upper	1.000			1.000		
Middle	0.8534	0.4595-1.585	0.596	0.934	0.507-1.720	0.826
Lower	1.097	0.6117-1.967	0.759	1.655	0.862-3.180	0.130
Histological grade						
1	1.000			1.000		
2	1.991	1.300-3.050	**<0.001**	2.252	1.436-3.534	**<0.001**
3	2.135	1.104-4.130	0.004	2.488	1.420-4.358	**0.001**
pT status						
T1/T2	1.000			1.000		
T3/T4	2.184	1.499-3.180	**<0.001**	1.530	0.966-2.423	0.070
pN status						
N0	1.000			1.000		
N1	1.757	0.9385-3.289	0.031	1.134	0.544-2.362	0.738
N2	3.265	1.429-7.459	**<0.001**	1.794	0.691-4.658	0.230
N3	4.968	1.409-17.52	**<0.001**	2.916	1.082-7.857	**0.034**
TNM stage						
Ⅰ	1.000			1.000		
Ⅱ	2.457	1.479-4.080	0.003	2.930	1.398-6.139	**0.004**
Ⅲ	4.876	2.765-8.599	**<0.001**	3.618	1.156-11.322	**0.027**
TSR						
stromal-low	1.000			1.000		
stromal-high	2.044	1.411-2.962	**<0.001**	2.531	1.657-3.867	**<0.001**

Bold indicates values with a significant difference *P*<0.05. HR: hazard ratio
