# Supplementary material for: The prognostic value of tumor-stromal ratio combined with TNM staging system in esophagus squamous cell carcinoma
Source: J Cancer. 2021 Jan 1;12(4):1105–14. doi: 10.7150/jca.50439 (PMC7797665; doi:10.7150/jca.50439)
Supplement: Supplementary file 1 — Supplementary figure 1. [file jcav12p1105s1.pdf]

Supplementary materials

Figure S1

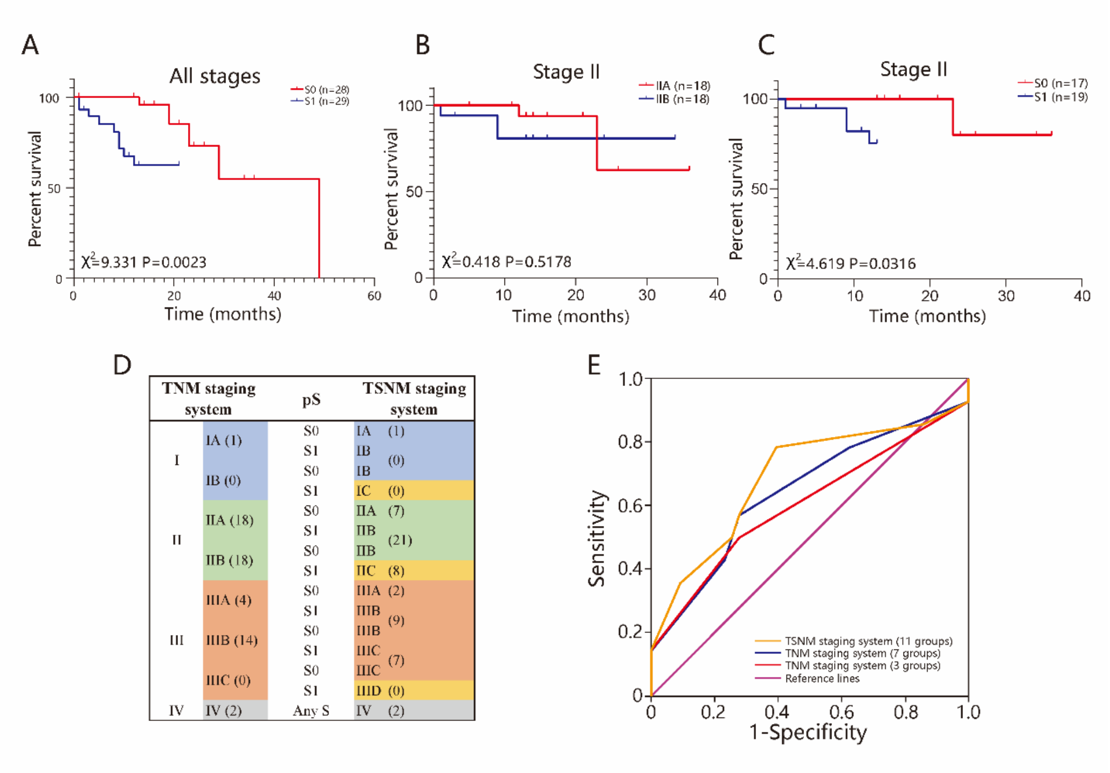

Figure S1 The combination of TSR and TNM staging system. A There was significant difference between S0 and S1 for all stages (*P*=0.0023). B Among the stage II, there was no significant difference between IIA and IIB (*P*=0.5178). C Among the stage II, there was significant difference between S0 and S1 (*P*=0.0316). D The TSNM staging system, based on 7th TNM staging system and pS status. The number represents the number of people who belong to the staging. E TSNM performed well in predicting the clinical outcomes of ESCC patients compared to other factors.
